# Unprecedented H5N1 outbreak: a rare cross-species influenza threat

**DOI:** 10.5713/ab.24.0560

**Published:** 2024-08-20

**Authors:** Cheol-Heui Yun

**Affiliations:** 1 Department of Agricultural Biotechnology, and Research Institute of Agriculture and Life Sciences, Seoul National University, Seoul 08826, Korea; 2 Co Editor-in-Chief, Animal Bioscience, Seoul 08776, Korea

**Keywords:** CDC Recommends, Cross-species Infection, H5N1, Highly Pathogenic Avian Influenza, HPAI

Highly pathogenic avian influenza (HPAI) H5N1, which is generally known to infect wild birds and poultry [1,2], was detected in an unusual and rare incidence of cross-species infection involving dairy cattle in Texas, underscoring ability of the virus to infect mammals. The virus that spreads in birds has already been documented to infect dozens of other mammalian species, although it seldom transmits between them [3]. The Minnesota Board of Animal Health reported on March 20, 2024 that a juvenile goat on a Minnesota farm tested positive for HPAI, marking the first instance of the virus in a domestic ruminant in the United States [4].

Many people believed for a long time that HPAI was incapable of spreading from birds through cows to humans although previous cases have shown that birds may. The degree of cow-to-cow transmission is unknown, and no human-to-human transmission has been identified. Cal-Maine Foods Inc., the largest egg producer in the United States, announced HPAI detection, at one of its Texas facilities, resulting in the depopulation of approximately 1.6 million laying hens and 337,000 younger hens, or roughly 3.6% of the company’s total flock [5]. It also recorded incidents in a Kansas facility earlier this year, “resulting in the depopulation” of nearly 1.5 million hens. Production at the facility has been temporarily suspended as the company adheres to the USDA guidelines [6].

While the influenza surveillance systems at the Centers for Disease Control and prevention (CDC) show no signs of unusual influenza activity in humans, including avian influenza A, there are growing concerns and public awareness about the H5N1 outbreak (Figure 1), which began in Texas (H5N1, Eurasian lineage goose/Guangdong clade 2.3.4.4b viruses) and a rare cross-species influenza threat. Cow-to-cow transmission is plausible because infections were observed in cattle on Michigan, Idaho, and Ohio farms where the virus-infected cows were transported [7]. It was discovered that the genomic sequences of the viruses from the three human cases on the poultry farm in Colorado were closely related to viruses found in recent poultry outbreaks and infected dairy cattle herds [8]. The sequences retain predominantly avian genetic characteristics and lack modifications that would make them more suitable for future human transmission. Although the exact source of contamination has not been identified, while H5N1 is not typically associated with milk transmission, theoretical possibilities for how it could invade and infect humans include contaminated milk from H5N1-infected due to dairy cattle, inadequate pasteurization, and direct contact when handling contaminated milk or dairy products. While transmission of the virus via milk is exceedingly unlikely under typical food safety practices, theoretical concerns exist if the milk has been contaminated or inadequately pasteurized. Needless to say, cautious zoonotic disease surveillance and strict adherence to food safety measures are critical for avoiding potential human infections (see Table 1 for CDC recommends).

The H5N1 has a similar life cycle to other viruses. It begins by binding to particular sialic acids found in birds and humans. The virus enters target cells by endocytosis, forming an endosome. The acidic environment inside the endosome causes changes in the viral hemagglutinin protein, leading it to fuse with the endosomal membrane. The viral ribonucleoprotein complexes are then released into the cytoplasm and transported to the nucleus, where viral RNA is transcribed and replicated. Finally, new virus particles form at the cell membrane and bud off, taking a portion of the host cell membrane as an envelope. The H5N1 predominantly infects respiratory epithelial cells in birds and mammals. In birds, the virus can infect gastrointestinal epithelial cells. In humans, however, the virus attacks cells in the lower respiratory tract, particularly alveolar epithelial cells, causing serious respiratory diseases and complications. The HPAI symptoms in humans include a high temperature or shivering, aching muscles, headache, and cough or shortness of breath, whereas in avian species it has resulted in abrupt death with no prior indicators, poor energy or appetite, reduced egg production, soft-shelled/misshapen eggs, or diarrhea. The HPAI in cattle causes a reduction in milk production and loss of appetite, making it difficult to identify.

The H5N1 outbreak in cattle in the United States should not lead us to panic because there have been few cases of human illness and no human-to-human transmission has been documented in connection with the outbreak. However, it is important to note that the worst-case scenario for HPAI infection in humans includes a high mortality rate with pandemic potential, as well as zoonotic potential with severe respiratory illness such as severe pneumonia, acute respiratory distress syndrome, and multi-organ failure.

Collectively, the recent detection of HPAI H5N1 in dairy cattle is a cross-species infection, underscoring the adaptability of the virus to infect mammals, particularly domestic ruminants across multiple farms. The outbreak has had a huge economic impact, including the depopulation of millions of hens. While current influenza surveillance indicates no unusual human influenza activity, the situation has raised public concerns due to the virus’s zoonotic potential and its devastating impact on both avian and mammalian species. Most importantly, thorough surveillance and adherence to food safety measures to prevent potential human infections are top priorities.

## Figures and Tables

**Figure 1:**
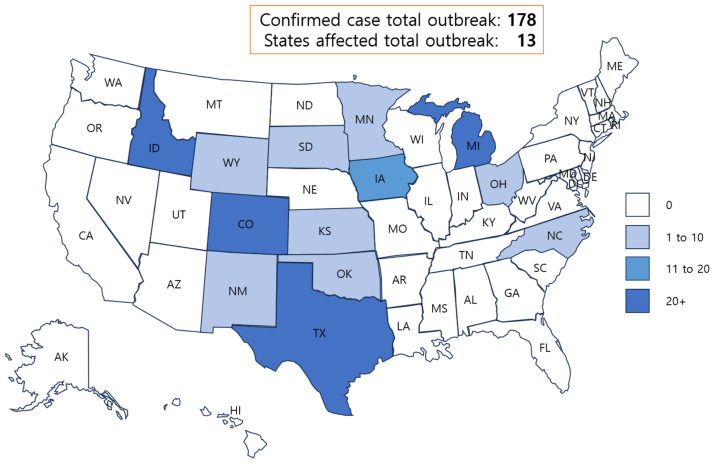
Confirmed cases of total outbreak and states affected in the USA [8] (as of Aug 02, 2024).

**Table 1:** CDC recommends

People should avoid exposures to sick or dead animals, including wild birds, poultry, other domesticated birds, and other wild or domesticated animals (including cows), if possible. People should also avoid exposures to animal poop, bedding (litter), unpasteurized (“raw”) milk, or materials that have been touched by, or close to, birds or other animals with suspected or confirmed avian influenza A(H5N1) virus, if possible. People should also avoid exposures to animal poop, bedding (litter), unpasteurized (“raw”) milk, or materials that have been touched by, or close to, birds or other animals with suspected or confirmed avian influenza A(H5N1) virus, if possible. People should not drink raw milk. Pasteurization kills avian influenza A(H5N1) viruses, and pasteurized milk is safe to drink. People who have job-related contact with infected or potentially infected birds or other animals should be aware of the risk of exposure to avian influenza viruses and should take proper precautions. People should wear appropriate and recommended personal protective equipment when exposed to an infected or potentially infected animal(s). CDC has recommendations for worker protection and use of personal protective equipment (PPE). CDC has interim recommendations for prevention, monitoring, and public health investigations of avian influenza A(H5N1) virus infections in people.

CDC A(H5N1) Bird Flu Response [Update August 2, 2024].

https://www.cdc.gov/bird-flu/spotlights/h5n1-response-08022024.html
